# Neutrophils in the era of immune checkpoint blockade

**DOI:** 10.1136/jitc-2020-002242

**Published:** 2021-07-21

**Authors:** Julien Faget, Solange Peters, Xavier Quantin, Etienne Meylan, Nathalie Bonnefoy

**Affiliations:** 1 IRCM, Inserm, Univ Montpellier, ICM, Montpellier, France, INSERM U1194, Montpellier, France; 2 Department of Oncology CHUV-UNIL, University Hospital Lausanne, Lausanne, Switzerland; 3 Service d'Oncologie Médicale, Institut régional du Cancer de Montpellier, 34298, Montpellier, France; 4 Swiss Institute for Experimental Cancer Research, EPFL, Lausanne, Switzerland

**Keywords:** programmed cell death 1 receptor, immunotherapy, active, tumor escape, myeloid-derived suppressor cells, neutrophil infiltration

## Abstract

The immune checkpoint blockade-based immunotherapies are revolutionizing cancer management. Tumor-associated neutrophils (TANs) were recently highlighted to have a pivotal role in modulating the tumor microenvironment and the antitumor immune response. However, these cells were largely ignored during the development of therapies based on programmed cell death receptor or ligand-1 and cytotoxic T lymphocyte antigen-4 immune checkpoint inhibitors (ICIs). Latest evidences of neutrophil functional diversity in tumor raised many questions and suggest that targeting these cells can offer new treatment opportunities in the context of ICI development. Here, we summarized key information on TAN origin, function, and plasticity that should be considered when developing ICIs and provide a detailed review of the ongoing clinical trials that combine ICIs and a second compound that might affect or be affected by TANs. This review article synthetizes important notions from the literature demonstrating that: (1) Cancer development associates with a profound alteration of neutrophil biogenesis and function that can predict and interfere with the response to ICIs, (2) Neutrophil infiltration in tumor is associated with key features of resistance to ICIs, and (3) TANs play an important role in resistance to antiangiogenic drugs reducing their clinical benefit when used in combination with ICIs. Finally, exploring the clinical/translational aspects of neutrophil impact on the response to ICIs offers the opportunity to propose new translational research avenues to better understand TAN biology and treat patients.

## Introduction

Immune checkpoint blockade is the latest revolution in the care of patients with cancer. Anticytotoxic T lymphocyte antigen-4 (CTLA-4) and antiprogrammed cell death receptor or ligand-1 (PD-1/PD-L1) antagonist antibodies, which work by inducing the expansion of type-I helper CD4^+^ T lymphocytes (Th1) or by restoring the antitumor activity of exhausted CD8^+^ T cells,^1 2^ are giving promising results (long-term survival increase) in a subset (25%–40%) of patients with melanoma, lung cancer, colorectal cancer (CRC),^3^ and urothelial carcinoma.^4^ However, important challenges to improve the clinical benefit of these therapeutics, globally designated as immune checkpoint inhibitors (ICIs), concern the identification of predictive markers and the characterization of resistance mechanisms.^5 6^ Major efforts are made to decipher the immunosuppressive molecular and cellular mechanisms in the tumor microenvironment (TME), to better stratify patients who will benefit from ICIs, and to identify new targetable pathways to enhance their antitumor immune responses. In this context, myeloid cells and particularly neutrophils have recently emerged as major immune contributors to cancer progression and resistance to ICIs.

Neutrophils are frequently identified as CD66b^+^, CD15^+^CD14^−^, CD33^+^ cells in humans and as CD11b^+^Ly6C^int^Ly6G^hi^ cells in mice.^7^ Although tumor-associated neutrophils (TANs) seem to be predominantly associated with bad prognosis and poor response to therapy in multiple solid tumor types,^8^ they also show some plasticity with important consequences on disease progression. Indeed, they can polarize toward tumor-promoting (N2-TAN) or antitumor (N1-TAN)^9^ cells in mice. Neutrophils in blood can be subdivided into normal-density neutrophils and low-density neutrophils (LDN) from gradient centrifugation. Immunosuppressive neutrophils from blood and tumors are also frequently designated as granulocytic-myeloid derived suppressor cells (G-MDSC) and more recently as polymorphonuclear-MDSCs (PMN-MDSC). In the most publications, PMN-MDSC and G-MDSC refer to a population of cells that express Ly6G in mice, CD66b and/or CD15 in humans able to inhibit T cell proliferation, cytokine secretion and cytotoxic activity in vitro. For a very detailed review on neutrophil markers in the context of cancer, see Jaillon *et al*.^10^ In mouse models of breast cancer, TANs can display protumor functions by reactivating dormant lung metastasis growth during inflammation.^11^ On the other hand, they can also have antitumor activity^9^ and protect against early stages lung cancer^12^ and breast cancer metastasis in mouse lung.^13^ Hence, TANs constitute a pool of cells with a broad range of activities and phenotypes. Their functional diversity is still poorly understood, but might be explained by signals coming from the TME, and probably also by other regulatory pathways involved in neutrophil biogenesis or differentiation.

We will see that in many circumstances, immunosuppressive and tumor-promoting neutrophils can negatively interfere with ICIs. To apprehend neutrophils biology in the context of ICI treatment, we decided to simplify the nomenclature of these cells by designating neutrophils present in the TME as ‘TANs’ and we use the term neutrophil for referring to cells present in the blood or the bone marrow.

Yet, TANs and their impact on the TME are usually ignored during the development and evaluation of ICI-based therapies, although they could help to explain the success or failure of many clinical trials. In this review, we propose that innovant therapies can affect TAN behavior, and consequently might deeply modify the care of patients with cancer by increasing ICI strength and success rate in the near future.

## Specific TAN effects and related tumor characteristics linking neutrophil with ICI response

**Figure 1:**
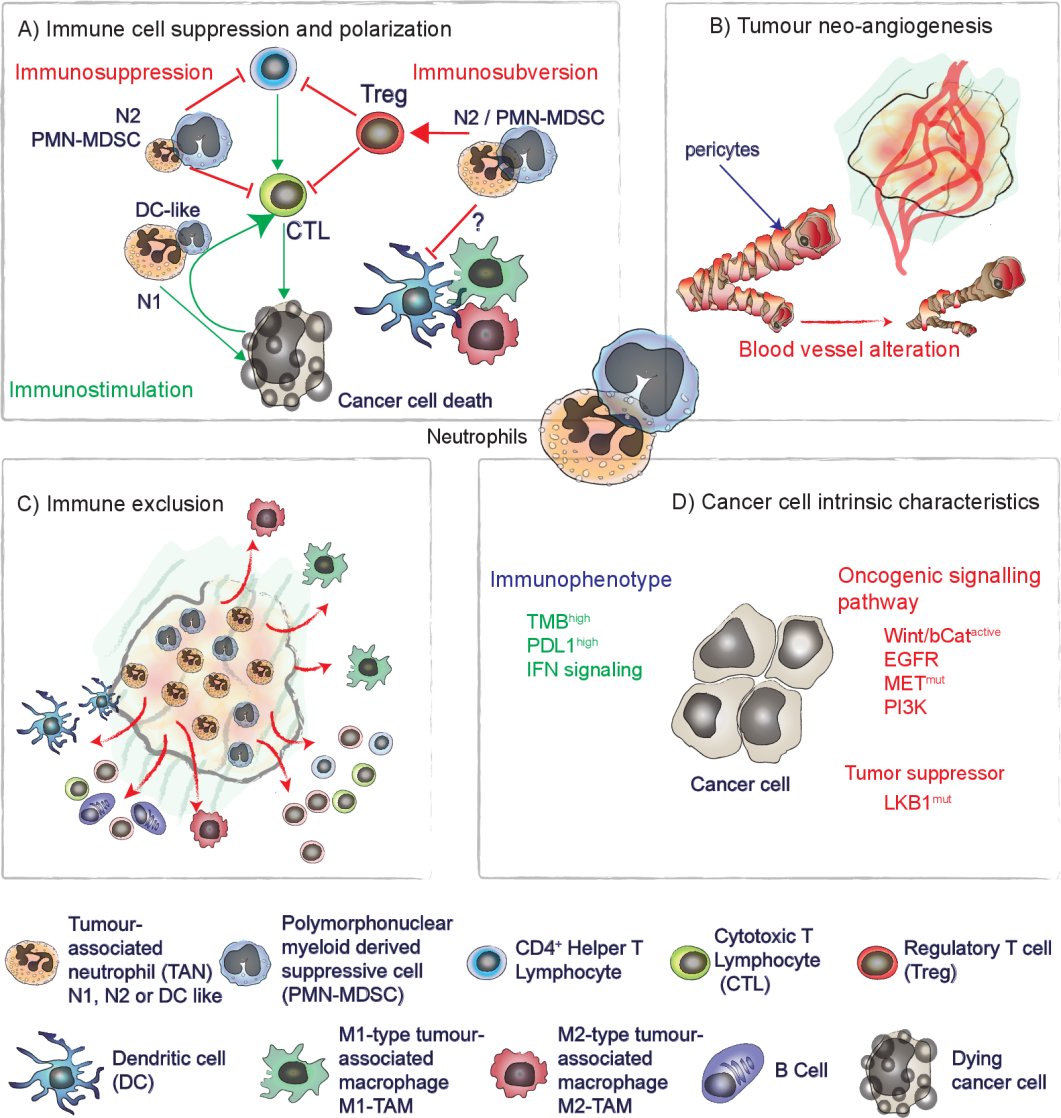
Specific TAN effects and related tumor characteristics linking neutrophil with ICI response. We identified four key features of the response to ICIs that are linked to neutrophil infiltration. (A) Immune cell suppression and polarization: TAN can inhibit T-cell functions in vitro and in vivo, favoring the recruitment of immunosuppressive Treg and altering dendritic cell and macrophage functions. (B) Tumor neoangiogenesis: neutrophils are associated with different tumor neoangiogenesis mechanisms and resistance to anti-VEGF antibody treatment in various preclinical settings. (C) Immune exclusion: neutrophil accumulation in the tumor mass is associated with reduced tumor infiltration by adaptive immune cells and macrophages, limiting ICI efficacy in various models. (D) Cancer cell intrinsic characteristics: strong TMB, expression of PD-L1 and integrity of IFN signaling are associated with a better response to ICIs. Conversely, mutations leading to hyperactivation of the Wnt/β-cat, EGFR, MET, and PI3K signaling pathways are associated with ICI resistance. MET and PI3K signaling alterations and loss of LKB1 have been associated with increased tumor-promoting neutrophil recruitment. (A, C) Can be seen as TAN-specific effect affecting ICI response while (B, D) are characteristics known to be linked with ICI response and related to TAN infiltration and tumor-promoting effect. EGFR, endothelial growth factor receptor; ICI, immune checkpoint inhibitor; IFN, interferon; PD-L1, programmed cell death ligand-1; PI3K, phosphatidylinositol-3-kinase; TMB, tumor mutational burden; Treg, regulatory T cell; VEGF, vascular endothelial growth factor.

Neutrophils and TANs are associated with key features of resistance to ICIs: (1) adaptive immune cell polarization and suppression, (2) tumor neoangiogenesis, (3) immune exclusion, and (4) cancer cell intrinsic characteristics (figure 1).

## TANs and tumor-infiltrating T lymphocytes

Comparison of different cell lines generated from the KPC pancreatic ductal adenocarcinoma mouse model confirmed that the cell lines forming PD-1^+^ CD8^+^ T cell-rich tumors are sensitive to combination treatments containing gemcitabine, paclitaxel, anti-CD40, anti-CTLA-4, and anti-PD-1 molecules. Conversely, tumor-infiltrating T lymphocyte (TIL)-poor tumors are resistant and contain a higher fraction of TANs. Abolishing CXCL1 or CXCL5/CXCR2 signaling in TIL-poor tumors reduces TAN infiltration, increases PD-1^+^ CD8^+^ T-cell abundance, and sensitizes tumors to the anti-CD40, anti-CTLA-4, and anti-PD-1 combination immunotherapy^14^ (table 1). Similarly in breast cancer, TAN rich tumors display lower macrophage and TIL infiltration and are resistant to anti-PD-1 treatment.^15^ Hence, the relationship between TANs and antitumor T cell response suggests the existence of a direct TAN-mediated suppression of Th1 and cytotoxic T lymphocytes in tumors. In this regard, arginase-1 (ARG1)-expressing human granulocytic cells leads to CD3ζ chain downregulation on T cells though L-arginine depletion and ultimately inhibit T-cell proliferation and cytokine secretion. In patients with non-small cell lung cancer (NSCLC), the presence of peripheral ARG1^+^ neutrophils increases with the tumor stage in treatment-naive patients and negatively correlates with the proportion of CD8^+^ T cells.^16^ Similarly, in patients with gastric cancer, a population of CD11b^low^CD33^low^CD66b^hi^ cells was identified in peripheral blood that suppress CD8^+^ cell activity.^17^ ARG1 expression has also been observed in peripheral CCR5^+^ LDNs from patients with melanoma. The proportion of such cells in peripheral blood mononuclear cells and their ARG1 expression increase with disease progression. It has been proposed that expression of CCL3, 4, and 5 in the tumor mass drives the recruitment of ARG1^+^CCR5^+^ LDNs^18^ in tumors. Interestingly, in the Kras^G12D^ mouse model of lung cancer, ARG1 inhibition reduces tumor growth and increases T-cell homing and function in tumors.^19^ Besides ARG1 expression, the release of prostaglandin-E2 (PGE2) by neutrophils and TANs represents another important mechanism of T-cell suppression. Very recently, it was reported that FATP2 expression plays a critical role in PMN-MDSC and TAN-mediated T-cell suppression. FATP2 is involved in triglyceride-containing arachidonic acid uptake by PMN-MDSC, and consequently, in the production of immunosuppressive PGE2 through its degradation by the prostaglandin E synthase (COX2). FATP2-deficient LDNs show lower ability to suppress T-cell amplification in vitro, and FATP2 inhibition using lipofermata reduces tumor growth and increases sensitivity to anti-CTLA-4 immunotherapy in the EL4, CT26, LLC, and TC-1 cancer cell-derived mouse models.^20^ TANs can also suppress T-cell proliferation and function, or induce their apoptosis through the production of reactive oxygen species (ROS) and nitric oxide (NO),^9 21^ Fas ligand,^22^ and TRAIL.^23^

**Table 1:** Clinical trials combining immune checkpoint blockade and drugs that may affect resistance linked with neutrophil biology

	Target	Drug	Immune checkpoint inhibitor	Clincal trial	Cancer type	Phase
	DPP8, DPP9, and FAP inhibitor	Talabostat	Pembrolizumab	NCT04171219	Solid neoplasm	Phase II
Biogenesis			Pembrolizumab	NCT03910660	Prostate cancer/neuroendocrine tumors/small cell carcinoma	Phase I/II
	IL6	Tocilizumab	Nivolumab, ipilimumab	NCT04258150	Pancreatic cancer	Phase II
			Nivolumab, ipilimumab	NCT03999749	Melanoma	Phase II
	STAT3	BBI-608 (napabucasin)	Pembrolizumab	NCT02851004	Colorectal cancer	Phase I/II
			Ipilimumab/nivolumab/pembrolizumab	NCT02467361	Cancers	Phase I/II
			Nivolumab	NCT04299880	Oncology	Phase I
			Nivolumab	NCT03047839	Colorectal cancer	Phase II
	IL1-beta	Canakinumb	Spartalizumab	NCT04028245	Renal cell carcinoma	Early phase I
			Spartalizumab	NCT03742349	Triple-negative breast cancer	Phase I
			Pembrolizumab	NCT03968419	Non-small cell lung cancer	Phase II
			Pembrolizumab	NCT03631199	Non-small cell lung cancer	Phase III
	RIP-1	GSK3145095	Pembrolizumab	NCT03681951	Advenced solid tumors	Phase II
Recruitment	LXR-alpha/beta	RGX-104	Nivolumab, ipilimumab, pembrolizumab	NCT02922764	Malignant neoplasms	Phase I
	CXCR4/CXCL12	BL-8040/BKT140	Pembrolizumab	NCT02907099	Metatstatic pancreatic adenocarcinoma	Phase II
			Pembrolizumab	NCT02826486	Metatstatic pancreatic denocarcinoma	Phase II
		AMD3100/plerixafor	Pembrolizumab	NCT04058145	Metastatic head and neck squamous cell carcinoma	Phase II
		X4P-001	Pembrolizumab	NCT02823405	Advenced melanoma	Phase I
			Nivolumab	NCT02923531	Clear cell renal cell carcinoma	Phase Ib/II
	CXCR4/CXCR7	NOX-A12/olaptesed	Pembrolizumab	NCT03168139	Metastatic colorectal cancer/metastatic pancreatic cancer	Phase Ib/II
	CXCR1/2	SX-682	Pembrolizumab	NCT03161431	Advenced melanoma	Phase I
		Navarixin	Pembrolizumab	NCT03473925	Non-small cell lung cancer/castration-resistant prostate cancer/MSS colorectal cancer	Phase II
	IL8	BMS-986253	Nivolumab	NCT04123379	Non-small cell lung cancer and HNCC	Phase II
	CCR2/5	BMS-813160	Nivolumab	NCT03184870	Metastatic colorectal cancer and pancreatic cancer	Phase I/II
				NCT04123379	Non-small cell lung cancer and HNCC	Phase II
	CCR5	vicriviroc	Pembrolizumab	NCT03631407	Advenced MSS colorectal cancer	Phase II
	C5aR	IPH5401	Durvalumab	NCT03665129	Non-small cell lung cancer/hepatocellular carcinoma	Phase I
PI3K inhibitors	PI3K alpha	Duvelisib	Pembrolizumab	NCT04193293	Head and neck small cell cancer	Phase I/II
	PI3K-beta	GSK2636771	Pembrolizumab	NCT03131908	Melanoma	Phase I/II
	PI3K alpha, beta, gamma, delta	Copanlisib/BAY80−6946/aliqopa	Nivolumab	NCT03502733	Solid tumor and lymphoma	Phase I
			Nivolumab	NCT03711058	MSS proficient solid tumors	Phase I/II
			Nivolumab	NCT03735628	Non-small cell lung cancer/head and neck small cell cancer/colorectal cancer/hepatocellular carcinoma	Phase I/II
			Durvalumab	NCT03842228	Solid tumors	Phase I
			Nivolumab±ipilimumab	NCT04317105	Solid tumors	Phase I/II
	PI3K delta/gamma and low affinity alpha/beta	Idelalisib / GS-1101/zydelig	Pembrolizumab	NCT03257722	Non-small cell lung cancer	Phase I/II
	PI3K-delta	Itacitinib / INCB050465/IBI376	Pembrolizumab	NCT02646748	Advanced solid tumors	Phase I
Immunosuppressive functions and inhibitory signaling	ARG1	INCB001158	Pembrolizumab	NCT02903914	Metastatic solidd tumors	Phase I/II
			Pembrolizumab	NCT03361228	Solid tumors	Phase I/II
		Pegzilarginase	Pembrolizumab	NCT03371979	Small cell lung cancer	Phase I/II
	COX2	Celecoxib	Nivolumab	NCT03864575	‘Cold’ solid tumors	Phase I/II
			Nivolumab+Ipilimumab	NCT03728179	Solid tumors	Phase I/II
	PGE2-receptor	Grapiprant/ARY-007	Pembrolizumab	NCT03696212	Non-small cell lung cancer	Phase I
				NCT03658772	MSS colorectal cancer	Phase I
	NOS	L-NMMA	Pembrolizumab	NCT03236935	Non-small cell lung cancer/head and neck small cell cancer/classical Hodgkin's lymphoma/urothelial carcinoma/bladder DNA repair-deficiency disorders	Phase I
	NOS	L-NMMA	Pembrolizumab	NCT04095689	Triple-negative breast cancer	Phase II
	CD47	ALX148	Pembrolizumab	NCT03013218	Metastatic cancer/solid tumor/advanced cancer/non-Hodgkin's lymphoma	Phase I
	SIRPa	TTI-621	Pembrolizumab	NCT02890368	Melanoma, Merkel-cell carcinoma/squamous cell carcinoma/breast carcinoma/HPV-related malignant neoplasm/soft tissue sarcoma	Phase I
			Nivolumab	NCT02663518	Haematologic/solid tumor	Phase I
		TJ011133/TJC4	Pembrolizumab	NCT03934814	Solid tumors/lymphoma	Phase I
	ILT4	MK-4830	Pembrolizumab	NCT04165083	Advenced solid tumors	Phase I

FAP, fibroblast activation protein; HNCC, head and neck cancer; MSS, microsatellite intability; PI3K, phosphatidylinositol-3-kinase.

More information on the real impact of these pathways may be revealed by ongoing clinical trials. Indeed, ARG1 inhibitors (INCB001158 and pegzilarginase) are currently tested in combination with pembrolizumab. Celecoxib (COX2 inhibitor) is evaluated in combination with nivolumab and nivolumab plus ipilimumab. The PGE2-receptor inhibitor grapiprant is also tested in combination with pembrolizumab (table 1). Finally, the NO synthase inhibitor monomethyl-L-arginine (L-NMMA) is assessed in combination with pembrolizumab and durvalumab (table 1). Of note, expression of iNOS and ROS2, which are involved in NO and ROS synthesis respectively, is not a strict marker of tumor-promoting TANs because both proteins can also participate in cancer cell killing by TANs.

In addition to the direct inhibition of effector T cell functions, TANs have also been implicated in regulatory T cell (Treg) recruitment. In KP mice, neutrophil depletion, using an anti-GR1 antibody or the combination of anti-Ly6G antibody and a secondary antirat antibody, has been associated with Treg reduction in lung tumors.^24 25^ Similarly, CCR5^+^ARG1^+^PDL1^+^ TAN infiltration is associated with increased Treg proportion in tumors from the human RET transgenic mouse model of melanoma. In this model, CCR5 inhibition reduces neutrophil recruitment in tumors and Treg proportion, and increases mouse survival.^18^ Finally, in the *Apc*^fl/fl^; *Cdx2*^CreERT2^ mouse model of CRC and in CRC samples from patients, TAN infiltration correlates with increased transforming growth factor-beta (TGFβ) signaling, whereas TAN depletion using the anti-GR1 antibody and CCR2 inhibitor combination results in increased T-cell homing in the tumor mass, characterized by a smaller proportion of Tregs. Interestingly, in this CRC model, TGFβ is mostly expressed by epithelial cells, monocytes and stromal cells, and the matrix metalloproteinase-9 (MMP9), which is abundantly expressed by TANs, converts pro-TGFβ into its active form. Consequently, inhibition of MMP9 and/or TGFβ receptor abolishes the neutrophil immunosuppressive and tumor-promoting functions.^26^

In conclusion, although TANs can exert a direct pressure toward TIL eradication through NO and ROS cytotoxicity, they can also suppress T-cell activation, cytokine secretion, and proliferation via L-arginine depletion or PGE2 production and drive Treg recruitment and perhaps expansion, establishing a T-antigen specific tolerance.

## TANs, tumor angiogenesis and immune exclusion

In the tumor mass, neoangiogenesis leads to the development of an immature microvascular network that shapes the TME architecture and composition^27 28^ and also contributes to disease progression and resistance to ICI treatment. TANs are considered a major source of growth factors involved in tumor angiogenesis, and their infiltration has been associated with resistance to antivascular endothelial growth factor (VEGF) therapy.^29^ The anti-VEGF and anti-angiopoietin-2 bispecific antibody venucizumab has been evaluated in a phase I clinical trial in combination with atezolizumab (anti-PD-L1 antibody) in patients with advanced cancer with acceptable safety profile and promising effect on tumor angiogenesis and cellular density were observed (NCT01688206). In pancreatic, breast, and brain mouse tumor models, treatment with anti-VEGFR and anti-PD-L1 antibodies together with a lymphotoxin-β receptor agonist led to the formation of high endothelial venules in tumors accompanied by cytotoxic lymphocyte infiltration.^30^ These findings linking angiogenesis to the TME have been reviewed elsewhere^31 32^ and suggest that the connections between TAN infiltration and tumor angiogenesis play an important role in ICI response.

We identified 111 clinical trials that are testing the combination of bevacizumab (Avastin; anti-VEGF-A antibody) with PD-1/PD-L1 or CTLA-4 blockade. Therefore, it is important to determine the role of TAN infiltration in the response to this strategy. Mouse neutrophils can release VEGF-A on CXCL1 stimulation through HCK and FGR Src tyrosine kinase signaling,^33^ but it is not known whether human neutrophils can also do it. Moreover, TAN negative impact on bevacizumab treatment remains poorly understood. In nude mice xenografted with human cancer cell lines (A-673, Calu-6, HM7, HPAC, Jurkat), Bv8/prokineticin-2 is upregulated in bone marrow neutrophils. The Bv8 soluble factor induces endothelial cell proliferation and angiogenesis through the activation of EG-VEGFR/PKR1 and EG-VEGFR/PKR2 signaling. Importantly, inhibition of Bv8 signaling using a specific antibody reduces CD11b^+^GR1^+^ myeloid cell mobilization in blood, TAN infiltration in tumors, and tumor angiogenesis in all tested models. Furthermore, in these models, Bv8 inhibition and anti-VEGF-A agents have a cumulative effect, demonstrating that both Bv8 and VEGF-A contribute to tumor neoangiogenesis.^34^ Considering TAN negative impact on bevacizumab efficacy, TAN infiltration should be specifically analyzed in clinical trials that combine ICIs and antibodies against VEGFR-2 (tanibirumab and ramucirumab), or VEGFR-2 inhibitors, such as apatinib. Multitargeted RTK inhibitors, such as pazopanib and sorafenib that inhibit PDGFR and VEGFR1–3 are currently tested in combination with ICIs. Importantly, sunitinib, which inhibits FGFR1–4, RET, PDGFR, KIT, CSF1R, and VEGFR1–2 could overcome neutrophil-mediated immunosuppression together with a simultaneous action on tumor angiogenesis and cancer cell oncogenic signaling.^35^ Evaluation of lenvatinib (that inhibits VEGFR1–3, FGFR1–4, KIT, RET, and PDGFR) plus pembrolizumab in a phase Ib/II clinical trial in NSCLC, renal cell carcinoma (RCC), melanoma, head and neck cancer (HNC), and urothelial carcinoma demonstrated good tolerability and promising therapeutic activity of this combination (NCT02501096).^36^ Similarly, anlotinib and pazopanib inhibit KIT and FLT3 signaling in addition to VEGFR, and thus, determining if these drugs could have a direct impact on neutrophils became particularly interesting. Indeed, all these tyrosine kinase inhibitor (TKI) could sensitize tumors to immune checkpoint blockade through their simultaneous action on tumor angiogenesis, cancer cell oncogenic pathways and neutrophils. However, combination of the anti-PD-1 nivolumab with pazopanib or sunitinib revealed the need of a precise dose and drug selection in these combinatory approaches (NCT01472081).^37^

**Figure 2:**
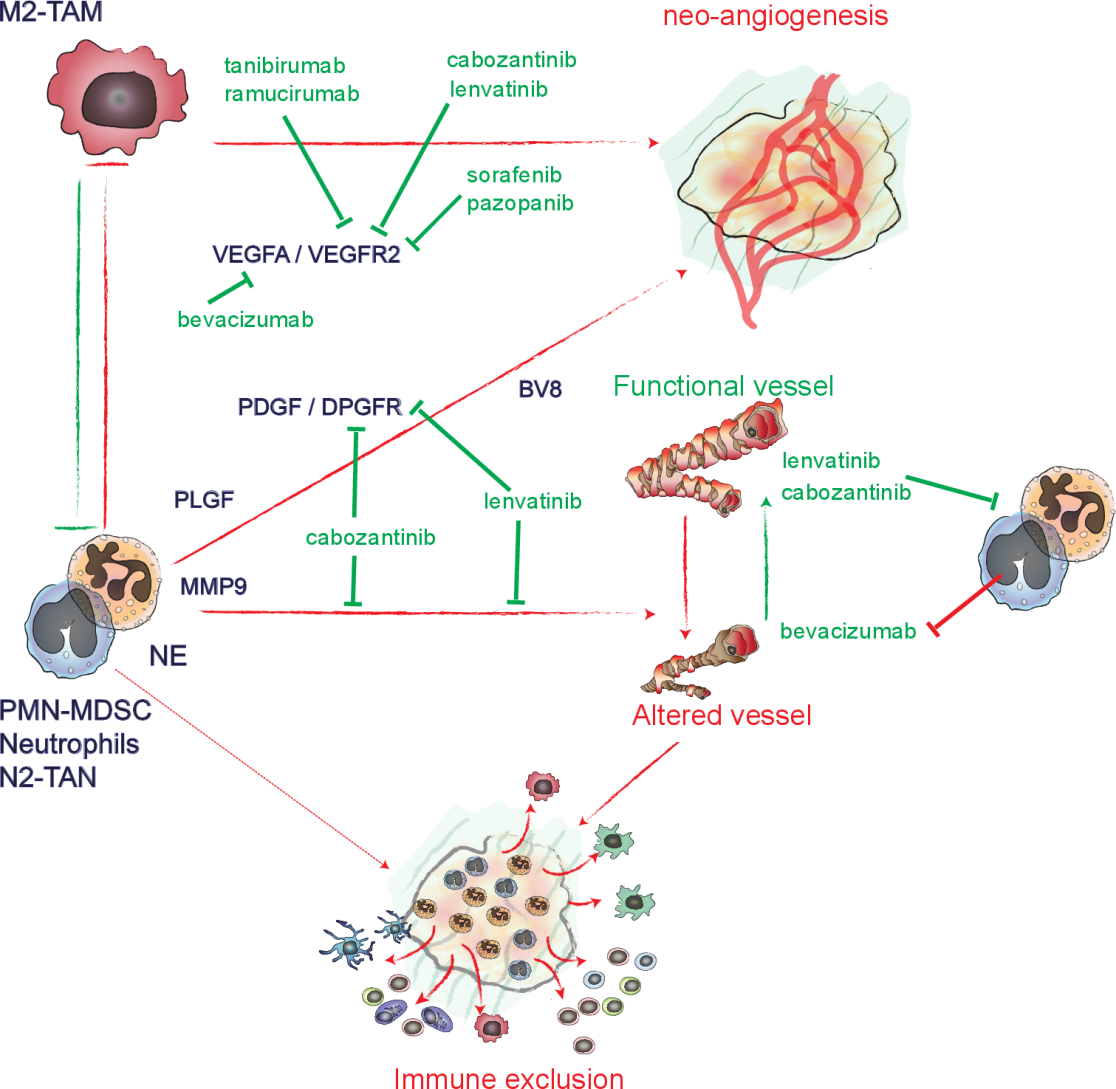
TAN, tumor angiogenesis, and therapeutics used in combination with ICIs TANs and macrophages play a key role in tumor neoangiogenesis. Tumors are highly infiltrated by macrophages or neutrophils, frequently in a mutually exclusive manner. However, in mouse models, these two cell types can substitute each other during neo-angiogenesis. Neutrophils secrete PlGF, PDGF, VEGFA, and Bv8 that directly contribute to neoangiogenesis. These cells also express MMP9 that plays an important role in neoangiogenesis. Neutrophil infiltration drives bevacizumab resistance, while the impact of this cell type on the response to anti-VEGFR2 antibodies (tanibirumab and ramucirumab) and small molecule inhibitors (sorafenib and pazopanib) remains to be characterized. Cabozantinib and lenvatinib, which block VEGFR1–3 and PDGFR and other tyrosine kinase receptors, inhibit neutrophil tumor-promoting function and neoangiogenesis. TANs promote the formation of an immature blood vessel network that lack pericyte coverage and that is not permissive to immune cell extravasation and recruitment in tumors. Ultimately, this contributes to immune exclusion. All the indicated drugs are currently tested in combination with ICIs. ICIs, immune checkpoint inhibitors; MMP9, matrix-metalloproteinase-9; PDGFR, platelet-derived growth factor receptor; PIGF, placental growth factor; TANs, tumor-associated neutrophils; VEGFA, vascular endothelial growth factor-alpha; VEGFR, VEGF receptor.

Study in the KP mouse model of lung cancer showed that neutrophil depletion (using an anti-GR1 antibody) increases tumor vasculature coverage by alpha-SMA^+^ pericytes, reduces hypoxia, reverts tumor immune exclusion and sensitizes tumor to anti-PD-1 therapy.^24^ In the CCR2-deficient mouse model of cervical cancer, tumor-associated macrophages (TAM) proportion is reduced compared with control tumors, and TANs support tumor angiogenesis in the absence of macrophages.^38^ Thus, when considering tumor angiogenesis, TAMs and TANs sometimes display overlapping functions through shared signaling mechanisms. Depending on the tumor models, infiltration can be predominantly by TAMs or TANs, and this will differentially affect the response to ICIs.^15 39^ Hence, identifying the molecular differences between TAM-related and TAN-related tumor angiogenesis may help to determine their specific contribution to tumor growth and to treatment response in different solid tumor types (figure 2).

## TANs and tumor cell intrinsic characteristics

The three major tumor-related diagnostic factors that are functionally associated with the response to ICIs are: (1) PD-L1 expression,^40 41^ (2) tumor mutational burden,^42^ and (3) specific oncogene/tumor-suppressor alterations in cancer cells.^43 44^

Specifically, the type of oncogenic signaling and tumor-suppressor gene alterations differently affects the response (resistance or sensitivity) to immune checkpoint blockade in a way that might involve neutrophils. For example, deletion of the tumor suppressor LKB1/*STK11* is associated with higher tumor-promoting TAN recruitment and immune checkpoint blockade resistance in lung cancer.^44 45^ The PTEN signaling pathway is also a great example of oncogenic pathway that links ICI resistance to TAN recruitment. In cancer cells, loss of PTEN is associated with phosphatidylinositol-3-kinase (PI3K)-AKT signaling overactivation that drives the expression of immunosuppressive cytokines, lowering T-cell recruitment, and enhancing TAN accumulation in the tumor mass.^46 47^ Therefore, PI3Kα/β inhibitors (duvelisib and GSK2636771) that target PTEN mutated cancer cells might also remodel the TME.^47 48^ Furthermore, the MET inhibitor capmatinib increases ICI efficacy in various solid cancer mouse models through alteration of neutrophil recruitment at sites with T cell-induced inflammation.^49^ A phase II clinical trial (NCT04139317) is currently testing the efficacy of pembrolizumab in NSCLC combined or not with capmatinib, irrespectively of the cancer MET mutation status. Finally, clinical innovations may come from cabozantinib, a MET, RET, AXL, VEGFR2, FLT3, and KIT inhibitor that reprograms TANs toward antitumor activity.^50 51^ There are currently 48 and 47 ongoing clinical trials in which the pan-TKI cabozantinib is combined with PD-1/PD-L1 or CTLA-4 blockade, respectively. Preliminary observations from the clinical trials with cabozantinib are promising, possibly due to the simultaneous targeting of cancer cell signal pathways (RET, MET, AXL), neutrophil function (MET, potentially KIT, and FLT3), and tumor angiogenesis (VEGFR2).^52^

## TAN diversity and recruitment as the cornerstone to understand ICI failure in solid tumors

While preexisting adaptive immune response in tumor is required for effective ICI response,^44 53^ TAN infiltration is predominantly associated with an adverse outcome across all cancer types^53^ and these cells present specific features linked with immunosuppression in tumors.^10 21^ Of note, approaches designed to inhibit neutrophil survival might trigger a compensatory neutrophil production in bone marrow, leading to the release of young neutrophils in the circulation, as our recent experiments in C56BL/6J mice treated with an anti-Ly6G antibody demonstrated.^25 54^ These newly generated neutrophils might develop opposite functions compared with those of mature neutrophils present in non-treated mice.^55^ Therefore, although neutrophil involvement seems obvious, it is often difficult to determine whether the observed biological effects are due to neutrophil number reduction (depletion) or increased renewal in tissues (turnover).^25 54^ Furthermore, association between neutrophils and response to ICIs is not restricted to the TME. Indeed, an alteration of neutrophil biogenesis and survival as reflected by an elevated absolute neutrophil count (ANC) in peripheral blood and an increased in the neutrophil-to-lymphocyte ratio (NLR) predicts the response to ICIs across multiple cancer types.^56 57^

## Neutrophil diversity in patients with cancer

TAN phenotypic diversity in cancer was formally demonstrated by single-cell transcriptomic analysis in samples from patients with lung cancer and from Kras^LSLG12D/WT^; p53^fl/fl^ (KP) mice with lung cancer.^58^ This study identified five populations of neutrophils in human tumors and six in mouse lung adenocarcinoma, and also six neutrophil clusters in blood samples from patients.^58^ Particularly, this analysis showed the existence of a subpopulation of human TANs characterized by the expression of peptidase inhibitor 3 (PI3).^58^ This cell subpopulation displays a similar transcriptional profile as the SiglecF^+^ tumor-promoting TANs identified earlier in KP mice.^59^ Interestingly, this tumor-specific neutrophil cluster identified in mouse lung tumor is also characterized by elevated expression of LOX1/*OLR1*, a potential marker of human LDN in peripheral blood of patients with cancer.^60^ Hence, similarity and differences between mouse and human tumor-promoting TANs remains to be clarified however the observation listed above suggest that both mice and human tumor promoting TANs might share, at least partially, similar transcriptomic identity and origin.

While immunosuppressive TAN and circulating neutrophils (frequently named PMN-MDSC or G-MDSC) are suspected to originate from immature neutrophils,^7^ in KP mice, tumor-promoting SiglecF^+^ TANs are mature, non-proliferating and long-lived cells that sediment in both low-density and high-density gradient layers.^61^ Furthermore, we have recently shown that maintenance of SiglecF^+^ tumor-promoting TAN infiltration requires metabolic adaptation of these cells that associates with Glut1 induction and Glut3 loss in the KP mouse model of lung cancer. Conditional knockout of Glut1 in neutrophils increases TAN turnover and reduces accumulation of SiglecF^+^ TANs in the TME delaying tumor growth and sensitizing them to radiotherapy.^62^ These observations emanating mostly from mouse models suggest that tumor-promoting neutrophils, as exemplified by SiglecF^+^ TAN and immunosuppressive neutrophils are referring to different types of cells, each of them contributing to disease progression and refractoriness to treatment.

Although TANs seem to be predominantly associated with bad prognosis and poor response to therapy in multiple solid tumor types, they also show some plasticity with important consequences on disease progression. Mirroring a concept initially developed to describe tumor-associated macrophages (TAMs), neutrophils were shown to polarize toward an antitumor phenotype (N1) in presence of interferon (IFN), by opposition of the acquisition of immunosuppressive and tumor-promoting functions (N2) in presence of TGFβ.^9^ However, usage of this denomination (N1 vs N2) became rare in latest publications and might not recapitulate the functional diversity of these cells in cancer patients. Nevertheless, TAN function was shown to be versatile in tumors ranging from antitumor to tumor-promoting activity along disease progression. TANs display antitumor activity at early stages of disease development through various mechanisms. In early-stages lung cancer TANs were shown to stimulate T-cell responses through the expression of the co-stimulatory molecules, prime tumor-antigen (T-Ag)-specific T-cell responses, and cross-present T-Ag to CD8^+^ T cells.^63^ In early-stages mouse model of uterine cancer neutrophils induce tumor cell detachment from the basement membrane reducing tumor growth,^64^ while in early-stage mouse model of CRC, neutrophils were proposed to restrict tumor-associated microbiota ultimately avoiding uncontrolled IL17-mediated inflammatory response and disease progression.^65^ Finally, tumor-suppressing neutrophils play a major role in trained immunity models. Indeed, mice treatment with β-glucan induces epigenetic modification of neutrophil progenitors and precursors driving the emergence of tumor suppressing neutrophils capable of controlling B16 melanoma tumor growth.^66^

These observations revealed that TAN diversity has to be taken in consideration while evaluating ICI treatments. Together with signals present in the TME, TAN diversity might also be orchestrated by a profound alteration of neutrophil biogenesis during cancer progression. Indeed, a study in the KP mouse model of lung cancer revealed that tumor cells remotely induce osteoblasts that regulate neutrophil biogenesis, leading to the accumulation of the SiglecF^+^ subpopulation of tumor-promoting TANs.^59^ Interestingly, the appearance of SiglecF^+^ neutrophils in a model of heart infraction was shown to be initiated in the bone marrow directly from neutrophil precursors,^67^ arguing that neutrophil plasticity during heart injury, as in tumor models, might originate from distal alteration of the neutropoiesis. Supporting a profound alteration of circulating neutrophil diversity in cancer patients, single-cell RNA sequencing comparing blood neutrophils from healthy donors and patients with NSCLC identified a cluster representing 8% and 40% of neutrophils in healthy donors and patients, respectively.^68^

**Figure 3:**
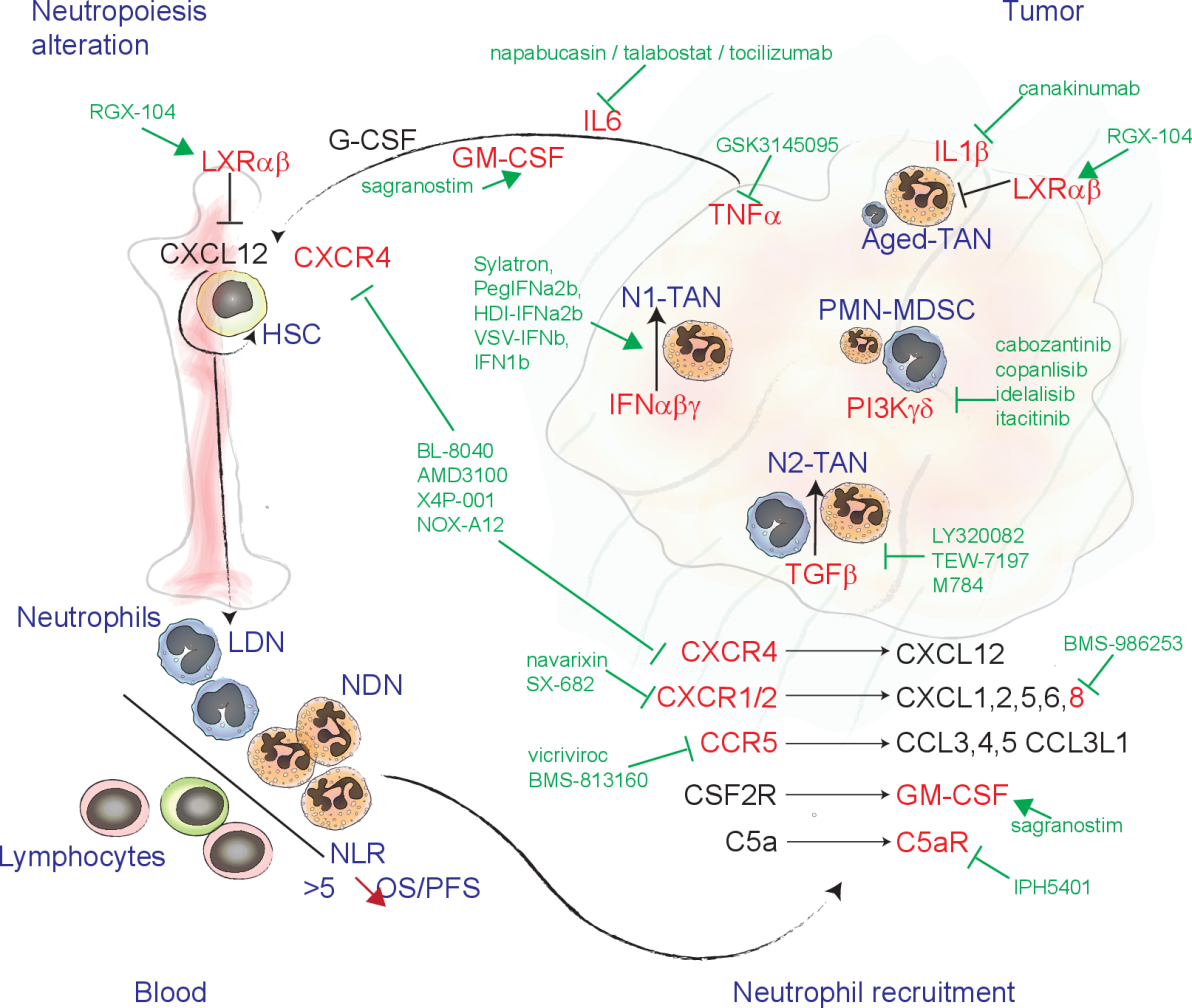
Drugs used in combination with ICIs that can affect neutrophil biogenesis and recruitment. Schematic representation of the targets (red) of drugs (green) currently combined with ICIs in clinical trials. Neutrophil biogenesis, during cancer development, is promoted by increased levels of TNFα, IL6, GM-CSF. The LXRαβ receptor plays a role in regulating CXCL12 expression in hematopoietic niches that govern HSC and neutrophil retention in bone marrow. In cancer patient peripheral blood, a population of LDN can accumulate, by opposition to NDN. The NLR is a strong predictor of OS and PFS in patients with cancer and treated by ICIs. neutrophils are recruited into the tumor mass through mechanisms that involve C-X-C motif chemokines and receptors, GM-CSF, and activated C5a. In the tumor, the balance between IFN and TGFβ signaling drives the acquisition of N1-TAN or N2-TAN functions. PIK3-γδ contributes to the accumulation of immature-like neutrophils, designated as PMN-MDSC, together with prosurvival signals, such as IL1β. C5a, complement component 5A; GM-CSF, granulocyte and macrophage colony stimulating factor; HSC, hematopoietic stem cells; ICI, immune checkpoint inhibitor; IFN, interferon; IL6, interleukin-6; LDN, low-density neutrophils; LXR, liver X receptor; NDN, normal-density neutrophils; NLR, neutrophil to lymphocyte ratio; OS, overall survival; PFS, progression-free survival; PIK3-γ, phosphatidylinositol-3-kinase gamma/delta; PMN-MDSC, polymorphonuclear myeloid derived suppressor cells; TAN, tumor-associated neutrophil; TGFβ, transforming growth factor-β; TNFα, tumor necrosis factor-α.

Hence, TAN accumulation and diversity in tumors are the result of neutrophil biogenesis alterations (for review, see Bergenfetz and Leandersson^7^), intratumor polarization, and prosurvival signals present in the TME as well as of active recruitment mechanisms, each of them offering actionable targets to reduce TAN negative impact on the response to ICIs (figure 3).

## NLR as a marker of the response to ICIs

The NLR, which is based on the determination of the ANC and absolute lymphocyte count (ALC) in peripheral blood of patients with cancer, is a strong non-invasive predictive marker of the response to ICIs. At baseline, high NLR (>2.5) has been associated with lower progression-free survival and lower disease-free survival in patients with NSCLC,^69–72^ small cell lung cancer,^73^ and melanoma^74 75^ treated by ICIs. Furthermore, ANC increase during anti-PD-1 immunotherapy is an independent predictive factor of rapid disease progression in patients with advanced gastric cancer.^76^ Conversely, in patients with RCC^77^ and NSCLC,^78^ a decrease of the NLR value at week six post-treatment with anti-PD-1/PD-L1 antibodies is associated with better objective response rate and overall survival (OS). As a strong evidence of the important predictive value of NLR in patients treated with ICIs, a study performed in 509 patients with advanced melanoma, NSCLC, RCC, HNC, bladder cancer, or sarcoma and treated with anti-CTLA-4 and/or anti-PD-1/PD-L1 antibodies found that pre-treatment NLR value <5 and a 16% to 28% decrease of the NLR value during treatment are associated with longer OS.^56^ Conversely, NLR increase during treatment correlates with shorter OS.^56^ Of note, NLR predictive value is not restricted to immune checkpoint blockade, but concerns also other antibody-based therapies, such as cetuximab (anti-EGFR antibody) and the anti-EGFR (cetuximab or panitumumab) and anti-VEGF (bevacizumab) antibody combination in patients with CRC.^79^ This is also true for conventional chemotherapy in patients with large-cell neuroendocrine lung cancer,^80^ hepatic cell carcinoma,^81 82^ RCC,^83^ and prostate cancer.^84^ While diminution of the ANC during treatment is associated with a better patient response to ICI in lung cancer,^85 86^ high baseline ALC associates with better response to ICI in HNC^87^ and patients with NSCLC.^88^ Hence, the NLR prognostic value comes from the cointegration of the ANC and ALC, which it is determined by biogenesis as well as survival of both neutrophils and lymphocytes (figure 3).

## Combining immune checkpoint blockade and inhibition of neutrophil recruitment in tumors

The expression of ELR^+^ C-X-C chemokines (CXCL1, 2, 5, 6, and 8) and their specific receptors CXCR1 and CXCR2 represent the main axis regulating neutrophil recruitment in inflamed tissues and tumors.^89–92^ In agreement, CXCR1/2 inhibitors (SX-682 and navarixin) significantly inhibit TAN accumulation in tumor tissues and increase the efficacy of anti-PD-1 immunotherapy in mice.^93^ These results might be translated into the clinic thanks to a newly developed anti-IL8/CXCL8 antibody (BMS-986253) currently tested in a phase II clinical trial (NCT04050462) in combination with nivolumab (anti-PD-1 antibody) in patients with advanced hepatocellular carcinoma (HCC).

More recently, the expression of the CCR5 ligands CCL3/MIP1α, CCL4/MIP1β, CCL3L1, and CCL5/RANTES also has been implicated in neutrophil recruitment to tumor tissues in patients with melanoma and mouse models as well as in patients with NSCLC.^94^ Therefore, several trials are assessing whether the combination of the CCR5 inhibitor vicriviroc with pembrolizumab (NCT03631407) or of the CCR2/5 inhibitor BMS-813160 with nivolumab (NCT03184870) could be more effective than immune checkpoint blockade alone by inhibiting TAN-mediated cancer promotion and immunosuppression.

TAN accumulation might be reduced also through inhibition of CXCR4 because it has been suggested that it directly contributes to neutrophil recruitment in the pre-metastatic niches in mice.^95^ Furthermore, CXCR4 inhibition could impair hematopoietic niches and neutrophil biogenesis by blocking CXCL12-mediated hematopoietic stem cell homing.^96^ Interestingly, the phase II clinical trial NCT02826486 recently demonstrated that the combination of BL-8040/BKT140 (a CXCR4 inhibitor) and pembrolizumab in patients with pancreatic cancer reduces TAN infiltration and increases cytotoxic T-cell function.^97^

Besides the classical chemotaxis pathways, members A and C of the IL-17 cytokine family contribute to neutrophil recruitment in various solid tumors mouse models.^98–103^ Hence, IL-17A secretion has been associated with granulocyte/macrophage-colony stimulating factor (GM-CSF)-dependent cancer neutropoiesis in mouse models.^100–102^ Although inhibiting IL-6 or GM-CSF signaling might interfere with IL-17A-related neutropoiesis and TAN infiltration, there is currently no therapeutic approach specifically targeting IL-17 signaling in combination with immune checkpoint blockade. Similarly to IL-17A secretion that links antibacterial immunity to the anti-cancer immune response in mice and human,^98 101^ release of the complement component 5-a (C5a) in the TME is another potent attractant for neutrophils and macrophages in mice and on purified human neutrophils in vitro.^104 105^ Although the impact of C5a receptor (C5aR) signaling on TAN plasticity and function remains unclear in cancer, the anti-C5aR antagonist antibody IPH5401 is currently tested in the combination with durvalumab anti-PD-L1 antibody (NCT03665129).

Finally, based on observations made in mice and humans, artificial activation of apolipoprotein E signaling using the LXR agonist RGX-104 is considered as one of the most promising approaches to inhibit neutrophil accumulation in tumors and in the periphery. This approach reduces TAN survival in mice and sensitize tumors to anti-PD-1 blocking antibodies.^106^ Furthermore, a phase I clinical trial (NCT02922764) showed that RGX-104 reduces the fraction of circulating neutrophils in CRC, RCC, sarcoma, uterine cancer, and melanoma.^106^ In addition to the direct effect on neutrophil survival, mouse models suggest that RGX-104 also alters neutrophil biogenesis through modulation of the hematopoietic niche function^107^ (figure 3, table 1).

## Targeting tumor-promoting TAN polarization together with ICI treatment

TAN functional diversity is partly the result of polarizing and survival signals that neutrophils receive while infiltrating the TME such as the balance between IFN and TGFβ^9^ signals, that drive N1 and N2 functions, respectively. However, whether neutrophil polarization towards the N1 phenotype may have a favorable impact in cancer therapy remains to be demonstrated. We identified 14 clinical trials in which ICIs are combined with type-I IFN (Sylatron, PegIFN2b, HDI IFNa-2b, VSV-IFNb, IFN1b), 3 trials that are testing ICIs with TGFβ signaling inhibitors (LY3200882, TEW-7197), and 11 clinical trials that use M7824, a bispecific antibody against TGFβ and PD-L1. These combined treatments might have a pleotropic activity on a broad range of immune populations, including TAN polarization towards the N1 phenotype.

The IL6/STAT3 pathway, which plays a pivotal role in cancer-induced inflammation, might also be involved in cancer-induced neutropoiesis and TAN recruitment.^108^ Indeed, IL6 production by tumor-associated fibroblasts in HCC induces PD-L1, CXCL8/IL8, TNFα, and CCL2 expression in TANs, and increases their survival and suppressive activity.^108^ Interestingly, ongoing trials are evaluating the potential synergistic effect of the fibroblast activation protein inhibitor talabostat, the anti-IL6 antibody tocilizumab, and STAT3 small molecule inhibitors, such as BBI-608/napabucasin, with ICIs, based on multiple mechanisms, including their expected effect on neutrophil biogenesis, homing, and survival^108^ (table 1).

The proinflammatory cytokine IL1β also might contribute to TAN accumulation, polarization, and survival in the TME.^109^ Here again, combining the anti-IL1β antibody canakinumab or the IL1R antagonist anakinra with ICIs in clinical trials might affect both IL1β-mediated cancer cell proliferation and TANs (table 1).

Similarly, tumor-necrosis factor (TNF) signaling inhibition could synergize with immune checkpoint blockade by directly influencing T cells and also by reducing IL-17 secretion and TAN recruitment. Indeed, RIP1 and/or RIP3 inhibition, which abolishes necrosome formation and TNF signaling, increases the antitumor activity of macrophages and reduces TAN recruitment in mouse models of pancreatic cancer.^110 111^ GSK3145095, a RIP1/RIP3 kinase inhibitor that might damp TAN-mediated tumor promotion, is currently evaluated in combination with pembrolizumab (anti-PD-1 antibody) in patients with pancreatic cancer (NCT03681951) (table 1). However, RIP1 and NF-kB can contribute to the induction of immunogenic cell death, supporting CD8^+^T cell cross-priming.^112^ In a model of soft tissue sarcoma, inhibition of IAP family antiapoptotic genes (cIAP1 and 2) that influence TNF and RIP1 signaling in cancer cells increases cancer cell death and favor antitumor immunity in a RIP1-dependent manner (for a review, see Annibaldi *et al*^113^). The IAP (cIAP, XIAP) inhibitor birinapan is now evaluated in combination with pembrolizumab. However, due to its effect on neutrophil recruitment and also on immunogenic tumor cell death in the presence of TNF signaling, more studies are required to determine whether RIP1 activation or inhibition should be privileged to enhance ICI efficacy.

Induction of the GM-CSF/CSF2R pathway activates STAT5 signaling, increasing survival and expression of PD-L1 and fatty acid transport protein 2 (FATP2) in neutrophils, ultimately contributing to TAN immunosuppressive functions in mice.^20^ However, the combination of sagramostim (recombinant GM-CSF) and ipilimumab (anti-CTLA-4 antibody), currently tested in phase I/II trials, increases OS in patients with melanoma from 12.7 to 17.5 months, compared with ipilimumab alone.^114^ This raised many questions on how GM-CSF influences TANs and other tumor-infiltrating immune cells when administered as an anticancer drug (figure 3).

The expression of ‘don’t eat me’ signals by cancer cells is a potent mechanism to inhibit tumor cell destruction by neutrophils. In cancer cells, CD47 expression and its interaction with its specific receptor, called signal-regulatory protein alpha (SIRPα), on the neutrophil membrane inhibits their destruction through trogoptosis.^115 116^ These data provided the rational for the current evaluation of the CD47 inhibitor ALX148 and anti-SIRPα (TTI-62) or anti-CD47 (TJC4) blocking antibodies in combination with ICIs. In the same way, immunoglobulin-like transcript-4 (ILT4, gene name *LILRB2*) is an inhibitory receptor that impairs neutrophil phagocytosis and respiratory burst,^117^ and the anti-ILT4 antibody MK-4830 is currently tested in combination with pembrolizumab (table 1). Inhibition of CD47/SIRPα and HLA-G/ILT4 signaling should increase TAN antitumor activity, phagocytosis, and cancer cell killing. However, clear mechanistic investigations in patients and mouse models are lacking to fully evaluate the impact of these treatments on neutrophil functions relative to the immune checkpoint blockade response.

Finally, the activation of the PI3K gamma/delta (PI3Kγ/δ) signaling in TANs might also play a major role during the acquisition of tumor promoting functions. In a mouse model of prostate cancer, treatment with the TKI cabozantinib or with the dual P13K/mTOR inhibitor BEZ235 reduces TAN infiltration and increases the response to the anti-CTLA-4 and anti-PD-1 antibody combination.^50^ Accordingly, anti-GR1 antibody-mediated neutrophil depletion enhances the response to immunotherapy in this model.^50^ Similarly, PI3Kδ/γ inhibition or anti-Ly6G antibody-mediated neutrophil depletion sensitizes mice with HNC to ICIs, while their combination (anti-Ly6G plus PI3Kδ/γ inhibition) does not further improve ICI efficacy demonstrating that PI3Kδ/γ acts through neutrophil functional modulation.^48 118 119^ Indeed, in the CT26 mouse model of CRC, which is characterized by massive TAN infiltration, inhibition of PI3Kδ/γ increases anti-PD-1 immunotherapy efficacy through impairment of TAN immunosuppressive functions.^118^ Copanlisib, which inhibits PI3Kα/β/γ/δ, and PI3kγ/δ-selective inhibitors (idelalisib, tenalisib, itacitinib) are promising drugs that can synergize with ICIs.

All together, these different approaches currently under evaluation in combination with ICIs should help decreasing tumor-promoting TAN polarization and survival and could simultaneously favor the emergence of antitumor neutrophils.

## Concluding remarks and perspectives

Among the 401 clinical trials based on ICI combination with a second drug that might influence or be influenced by TAN and neutrophil biology, 329 (82.45%) want to inhibit tumor vasculature function, and among them, 111 (27.7% of total) are based on VEGF inhibition with bevacizumab. As TANs contribute to bevacizumab resistance, their study becomes highly relevant for such combination settings. Furthermore, 23 (5.7%) of these clinical trials are testing drugs that could revert TAN immunosuppressive functions, and 48 (11.9%) are assessing strategies that might change neutrophil homing and polarization in tumors. This highlights that despite their major impact on the immune response in many solid cancer types, much remains to be investigated to increase the immune checkpoint blockade success through simultaneous TAN targeting. Studying TAN-mediated immune exclusion and immunosuppression implies important efforts to characterize tumor angiogenesis and blood vessel maturation in TAN-infiltrated cancer models.

An important emerging aspect of TAN biology is connecting neutrophil extracellular traps (NETs) with ICI response. Indeed, in patients with CRC^120^ and in mammary and lung cancer-bearing mice, peripheral blood neutrophils display increased susceptibility to NET formation, which might contribute to cancer-related lung thrombosis.^121^ Furthermore, NETs have been implicated in metastatic outgrowth in patients with liver cancer after surgical stress through induction of HMGB1/TLR9 signaling in cancer cells.^122^ Similarly, neutrophil elastase and MMP9 released in NETs on lipopolysaccharide-induced inflammation have been shown to reactivate dormant cancer cells in models of lung metastatic colonization.^11^ Studying ICI impact on NET formation will most probably help to identify new mechanisms linked to treatment refractoriness, hyperprogression, and might explain some deleterious side effects of immune checkpoint blockade. Indeed, PAD4 inhibitors that abolishes NET formation sensitized the 4T1 breast cancer cell line transplantable model of mammary tumor to anti-PD-1 plus anti-CTLA-4 treatment. The authors suggested that NETs might prevent interactions between tumor cells and cytotoxic effector immune cells.^123^ Similarly, in a pancreatic cancer model, IL17A signaling inhibition abolished neutrophil recruitment and NET formation in tumors. Inhibition of PAD4 phenocopied IL17A blockade by supressing NET formation, reverting CD8 T cell exclusion form the tumor mass and sensitizing to anti-PD-1 plus anti-CTLA-4 treatment^124^ (for review, see Treference 125).

To conclude, this review of the literature and of ongoing clinical trials raises awareness about the potentially strong impact of neutrophils on the response to ICIs, which should be considered with great attention. Inhibiting TAN tumor-promoting or tumor-protecting functions may substantially increase the success rate of immune checkpoint blockade and the spectrum of patients that can respond to it.

From that, we identified four key questions that should be addressed in the coming years to better understand and exploit neutrophil/TAN biology in the context of ICI development:

- What are the specificity of TAN-related tumor angiogenesis? Understanding how TANs affect tumor vasculature could be remarkable considering immune cell extravasation, drug diffusion and response to antiangiogenic therapy.
- In patients with cancer and in mouse tumor models, TAN or TAM recruitment seems to be mutually exclusive and have a differential impact on the response to ICIs. Are there any specific characteristics of cancer cells that could explain why TAN infiltration is favored in some tumor types rending them resistant to ICIs?
- Can neutrophils and TANs reinforce ICI-induced antitumor immune response though intrinsic properties (exacerbation of IFN signaling, cancer cell opsonization by anti-PD-L1 antibody, trogoptosis, tumor antigen presentation and TIL priming) and conversely could they directly be involved in immune-related adverse events and hyperprogression phenomena?
- What are the mechanisms governing TAN and neutrophil diversity in tumors? Can these mechanisms be therapeutically exploited to abolish TAN tumor-promoting functions and favor antitumor TAN activity?
